# Spontaneous Platelet Aggregation in Blood Is Mediated by FcγRIIA Stimulation of Bruton’s Tyrosine Kinase

**DOI:** 10.3390/ijms23010076

**Published:** 2021-12-22

**Authors:** Rundan Duan, Luise Goldmann, Ya Li, Christian Weber, Wolfgang Siess, Philipp von Hundelshausen

**Affiliations:** 1 Institute for Cardiovascular Prevention (IPEK), Ludwig-Maximilians-University (LMU), 80336 Munich, Germany; Rundan.Duan@med.uni-muenchen.de (R.D.); goldmann.luise@web.de (L.G.); Ya.Li@med.uni-muenchen.de (Y.L.); Christian.Weber@med.uni-muenchen.de (C.W.); Philipp.von_Hundelshausen@med.uni-muenchen.de (P.v.H.); 2 German Centre for Cardiovascular Research (DZHK), Partner Site Munich Heart Alliance, 80336 Munich, Germany

**Keywords:** platelet, FcγRIIA, Btk, PF4, CD32, spontaneous platelet aggregation, multiple electrode aggregometry, ibrutinib, fenebrutinib, GPVI

## Abstract

High platelet reactivity leading to spontaneous platelet aggregation (SPA) is a hallmark of cardiovascular diseases; however, the mechanism underlying SPA remains obscure. Platelet aggregation in stirred hirudin-anticoagulated blood was measured by multiple electrode aggregometry (MEA) for 10 min. SPA started after a delay of 2–3 min. In our cohort of healthy blood donors (*n* = 118), nine donors (8%) with high SPA (>250 AU*min) were detected. Pre-incubation of blood with two different antibodies against the platelet Fc-receptor (anti-FcγRIIA, CD32a) significantly reduced high SPA by 86%. High but not normal SPA was dose-dependently and significantly reduced by blocking Fc of human IgG with a specific antibody. SPA was completely abrogated by blood pre-incubation with the reversible Btk-inhibitor (BTKi) fenebrutinib (50 nM), and 3 h after intake of the irreversible BTKi ibrutinib (280 mg) by healthy volunteers. Increased SPA was associated with higher platelet GPVI reactivity. Anti-platelet factor 4 (PF4)/polyanion IgG complexes were excluded as activators of the platelet Fc-receptor. Our results indicate that high SPA in blood is due to platelet FcγRIIA stimulation by unidentified IgG complexes and mediated by Btk activation. The relevance of our findings for SPA as possible risk factor of cardiovascular diseases and pathogenic factor contributing to certain autoimmune diseases is discussed.

## 1. Introduction

High platelet reactivity leading to spontaneous platelet aggregation (SPA) is a hallmark of cardiovascular diseases; however, the mechanism underlying SPA remains obscure. SPA measured in platelet-rich plasma (PRP) or blood is enhanced in patients with diabetes, acute coronary syndrome, myocardial infarction, and cerebrovascular disease [1,2,3,4,5,6], and has been reported to be a predictive risk marker of arterial occlusions in patients with diabetes and myocardial infarction [7,8,9]. Measurement of SPA using blood instead of PRP may reflect better systemic platelet reactivity in vivo, since blood contains other blood cells modifying platelet reactivity, and also the whole heterogeneous platelet population including the larger, more reactive young reticulated platelets [10,11]. 

Platelet aggregation in stirred anticoagulated blood can be easily and reliably measured by multiple electrode aggregometry (MEA) [12], a method that is now widely used to determine stimulated platelet aggregation in blood in patients with various diseases. However, in stirred or shaken blood, ADP leaking along with ATP from red cells elicits SPA and enhances stimulus-induced platelet aggregation [13,14,15]. We found that the omission of stirring during the 3 min warming-up phase before start of the MEA measurement virtually eliminated the ATP/ADP release from red cells and the subsequent ADP-mediated SPA [15]. Using this protocol, we now describe in the present study our observations of a novel type of SPA in blood and delineate the underlying mechanism. 

## 2. Results

**Figure 1:**
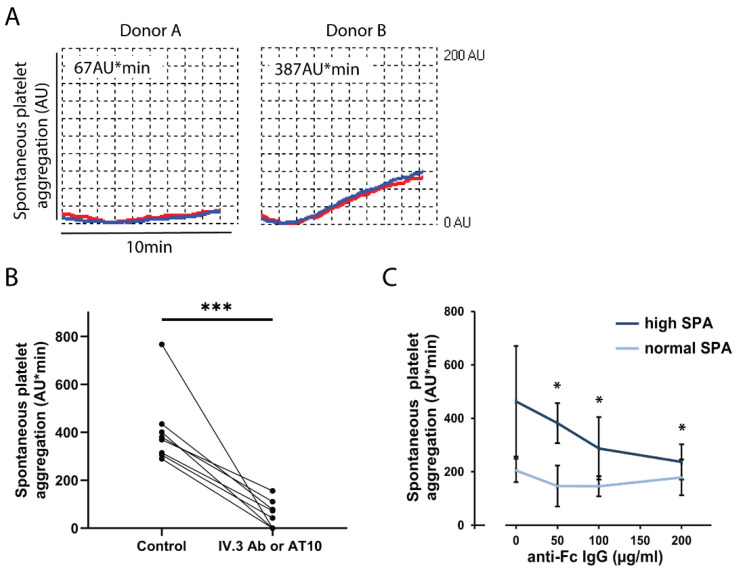
**Increased spontaneous platelet aggregation (SPA) in blood is mediated by activation of FcγRIIA.** (**A**) Normal and increased SPA in blood from donor A and B, respectively. The anticoagulated blood samples were prewarmed for 3 min without stirring at 37 °C. The stirrer was then added, and the measurement was started. Platelet aggregation was measured for 10 min by MEA. Representative tracings recorded by the two electrode pairs (blue, red) are shown. Increased SPA in donor B begins after 2 min. (**B**) Inhibition of increased SPA by the anti-FcγRIIA antibody IV.3 or AT10. Blood samples from subjects (*n* = 8) with increased SPA (289-767 AU*min) were incubated for 3 min without (control) or with the CD32 antibody IV.3 or AT10 (2 µg/mL), and then stirred for 10 min. The values are means ± SD (*n* = 8). *** *p* < 0.001. **C.** Effects of anti-human Fc-IgG antibody on SPA. Blood samples from donors with increased SPA (>250 AU*min, *n* = 4) and normal SPA (<250 AU*min, *n* = 4) were pre-incubated for 3 min with different concentrations (50, 100 and 200 μg/mL) of a goat antibody (IgG) directed against the Fc part of human IgG. Then, stirring and measurements (10 min) were started. The values are means ± SD (*n* = 4). * *p* < 0.05 compared to the samples without Fc-IgG antibodies.

Figure 1A shows representative aggregation tracings of a donor with high SPA as compared to a donor with normal SPA. SPA in blood from donor B began to increase about 2 min after start of stirring. By defining an aggregation threshold of >250 AU*min we detected in our pool of healthy blood donors (*n* = 118) 9 donors (8%) with increased SPA on 2 different days of measurements (duplicate or triplicate determinations) with intervals of 2 days to 22 months between the tests. In another seven donors, increased SPA was observed in only one out of two to three different experiments performed 7 days to 28 months apart. Thus, increased SPA could be found in some blood donors over a long time period. 

Next, we searched for the underlying mechanisms. Increased SPA was completely inhibited by pre-incubating the blood samples with the integrin αIIbβ3 blocking peptide RGDS (2 mM) or the anti-integrin αIIbβ3 antibody abciximab (20 µg/mL), indicating an essential role of the integrin αIIbβ3 (data not shown). Then, we used a large range of inhibitors to search for platelet receptors that might signal to activate the integrin αIIbβ3 mediating SPA. The inhibitors were: aspirin, the P2Y12 antagonist ticagrelor, ketanserin (5-hydroxytryptamine 2A receptor antagonist), lysophosphatidic acid receptor-5 (LPA5) receptor antagonists, hirudin in very high concentrations to ensure complete inhibition of thrombin and subsequent fibrin formation, the anti-GPVI antibody 5C4, GPVI-Fc, or the anti-FcγRIIA (CD32a) antibodies IV.3 and AT10. 

We found that only pre-incubation of blood samples with the anti-FcγRIIA antibodies significantly inhibited the increased SPA; the mean reduction was 85.9% (Figure 1B). FcγRIIA is typically activated by IgG immune complexes which bind with their Fc-part to FcγRIIA, thereby cross-linking the receptor and inducing intracellular signaling. Blood samples were therefore pre-incubated with increasing concentrations of goat anti-human Fc (IgG) antibody. High SPA was dose-dependently and significantly reduced by the anti-human Fc antibody; values of normal SPA were, however, not affected. This indicates that in certain blood donors the ligation of FcγRIIA by endogenous IgG immune complexes evokes high SPA.

**Figure 2:**
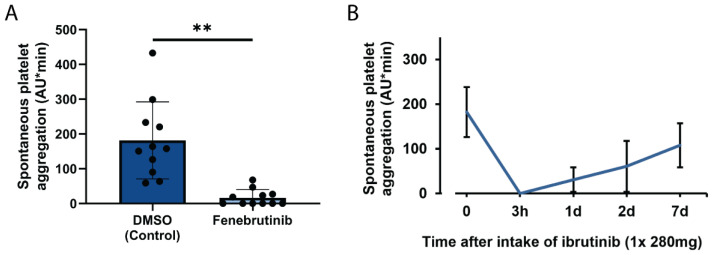
**SPA in blood is inhibited by Btk-inhibitors in vitro and ex vivo.** (**A**) Effect of the reversible Btk inhibitor fenebrutinib on SPA in vitro. The blood samples were incubated for 15 min with the solvent control DMSO (0.1%) or with fenebrutinib (50 nM) in the MEA cuvettes, then the stir bar was added, and the measurement (10 min) was started. The values are mean ± SD (*n* = 11). ** *p* < 0.01. The SPA values ranged from 59 to 432 AU*min. (**B**) Effect of oral intake of ibrutinib by a healthy blood donor on SPA ex vivo. SPA was measured before, and 3 hours (h), 1, 2, and 7 days (d) after intake of Imbruvica^®^ (280 mg). Representative results of one of three donors are shown. The values are means ± SD of triplicate determinations.

Stimulation of FcγRIIA leads to stimulation of Bruton tyrosine kinase (Btk) [16], and we had recently found that FcγRIIA-induced platelet aggregation, dense and α–granule secretion in blood is abrogated by Btk-inhibitors (BTKi) [17]. Pre-incubation of blood with the reversible BTKi fenebrutinib (50 nM) significantly inhibited SPA in donors with normal and high SPA values (59-432 AU*min) (Figure 2A). In addition, oral intake of a single dose of the irreversible BTKi ibrutinib (280 mg) by healthy volunteers completely inhibited SPA three hours after ingestion (Figure 2B). SPA inhibition was sustained for 2 days, which is explained by the irreversible, covalent Btk inactivation by ibrutinib and the lack of de novo protein synthesis in platelets. The recovery of SPA paralleled the expected physiological platelet renewal rate, and SPA was reversed toward control 7 days after ibrutinib intake. The results of inhibition of SPA by BTKi are in accordance with previous observations of SPA inhibition in blood by pre-incubating blood with various irreversible BTKi in vitro or by oral intake of ibrutinib ex vivo, either by CLL patients or by healthy volunteers [18]. 

**Figure 3:**
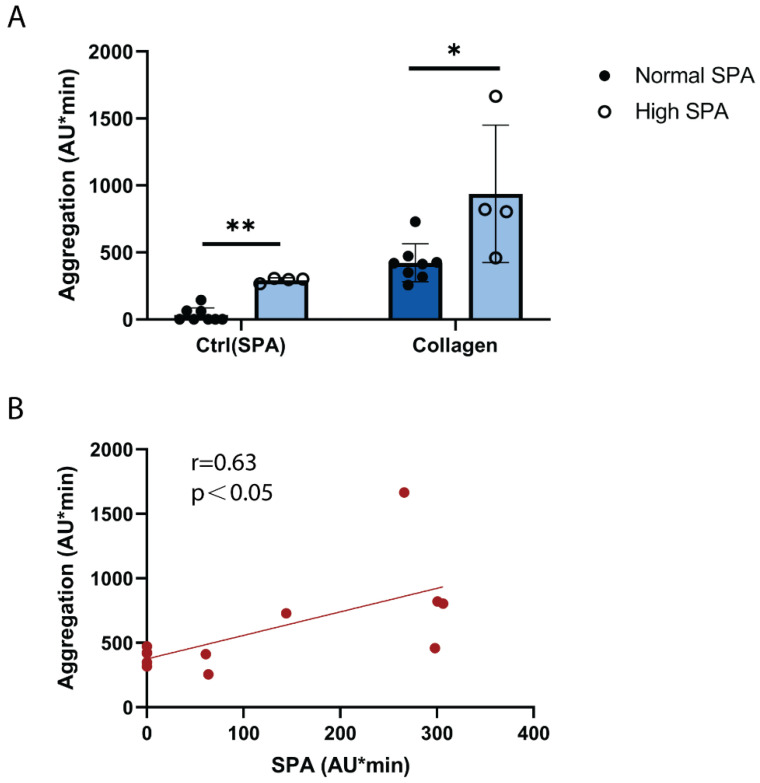
**High SPA is associated with increased platelet GPVI reactivity**. Hirudin-anticoagulated blood was prewarmed at 37 °C for 3 min. Collagen (0.1 µg/mL) was added, stirring was started, and platelet aggregation was measured for 10 min. (**A**) Blood of donors with high SPA responded more strongly to low-dose collagen than donors with normal SPA. Values are mean ± SD (*n* = 4 with high SPA and *n* = 8 with low SPA). * *p* < 0.05, ** *p* < 0.01. (**B**) SPA and platelet reactivity to collagen (0.1 µg/mL) correlate positively and significantly (Spearman correlation coefficient r = 0.63; *p* = 0.03). Each data point represents an individual subject (*n* = 12).

In order to explore whether SPA might affect platelet aggregation in response to stimuli, platelet aggregation was stimulated by a low concentration of collagen, which induces a low degree of platelet GPVI activation similar to atherosclerotic plaque homogenate [19,20]. By comparison with donors exhibiting normal SPA, donors with increased SPA showed a significantly increased platelet aggregation in response to collagen (Figure 3A). Furthermore, Figure 3B shows a significant positive correlation (r = 0.63, *p* ˂ 0.05) between SPA and platelet aggregation in response to low collagen concentration. These data provide a link of SPA and enhanced platelet reactivity to GPVI that both have been observed in patients with cardiovascular diseases (see Section 3). 

We also tested the platelet aggregation responses to the weak G-protein receptor coupled stimulus CXCL12 (SDF-1), a platelet chemokine involved in atherothrombosis [21]. However, no differences of platelet aggregation in donors with normal and high SPA and no correlation of SPA and CXCL12 induced aggregation were found (data not shown).

**Figure 4:**
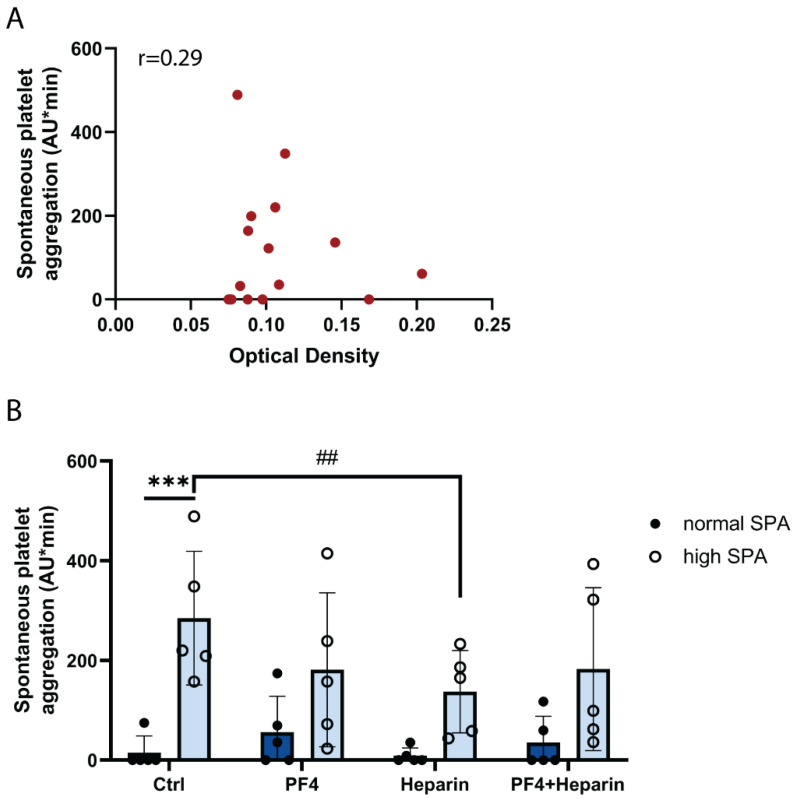
**SPA is not mediated by anti-PF4 IgG and not stimulated by PF4 and/or heparin.** (**A**) No correlation between SPA and serum titers of anti-PF4/polyanion IgG. The scatterplots display SPA and optical density of the IgG titers. Each dot on the graph represents a subject (*n* = 17). (**B**) SPA was not increased by incubation with PF4 and/or heparin. The hirudin-anticoagulated blood samples were prewarmed for 3 min at 37 °C. PF4 (5 µg/mL) or heparin (1 U/mL) were added, stirring was started and aggregation was measured for 10 min. The values are means ± SD (*n* = 5). *** *p* < 0.001, ## < 0.01.

To identify the platelet Fc-receptor activating IgG, antibodies to platelet factor 4 (PF4) in complex with polyvinylsulfonate (a heparin analogue) were measured by ELISA. Platelet factor 4 (PF4) is a positive-charged chemokine, and when binding to polyanions, the conformational change of PF4 is able to induce the generation of IgG antibodies that are able to cross-link and activate FcγRIIA receptors on the platelet surface [22]. Indeed, high titers of anti-PF4/polyanion and anti-PF4/heparin IgG are found in autoimmune heparin-induced thrombocytopenia (HIT) and vaccine-induced immune thrombotic thrombocytopenia (VITT) that stimulate platelet FcγRIIA receptors and subsequent Btk-mediated platelet activation [17,23,24,25,26,27]. Moreover, 5 to 7% of healthy blood donors have detectable PF4/heparin antibodies [28]. However, our data showed only very low anti-PF4/polyanion IgG titers with no correlation to SPA (correlation coefficient = 0.29; Figure 4A). Moreover, the addition of PF4, heparin or PF4 plus heparin did not increase SPA (Figure 4B). These data show that anti-PF4 IgG is not involved in triggering SPA.

## 3. Discussion

In the present study, we describe a novel protocol to measure SPA that uses stirred hirudin-anticoagulated blood and MEA, a device that had first been validated in 2006 by comparison with the single platelet counting (SPC) method that measures the decrease in platelet counts upon platelet aggregation [12]. MEA is now widely used to measure platelet function in patients with various hematological and cardiovascular diseases. SPA in previous studies was mostly measured in stirred, citrated anticoagulated PRP using light transmission or laser scattering methods [1,4,5,6,7,8,9]. However, the use of PRP for SPA measurement as compared to blood has several disadvantages: (a) other blood cells which can influence platelet reactivity are absent in PRP; (b) the preparation of PRP requires blood centrifugation that will per se and through secondary mechanisms modify platelet reactivity [1,12], and (c) will, dependent on the centrifugation speed, eliminate the larger and more reactive platelet populations. Furthermore, the use of citrate as anticoagulant favors platelet secretion and reactivity [29]. In the present study, hirudin was used as an anticoagulant, which does not alter the physiological blood concentrations of free Ca and Mg crucial for platelet aggregation [12,29]. In other previous studies, SPA was measured in shaken or stirred citrate-anticoagulated blood by SPC [2,3,30,31]. Interestingly, enhanced SPA was found in blood [2,3,30,31] but not PRP of diabetic patients [30] and was found to be related to the red blood cell fragility of diabetic patients [30]. Indeed, it is now known that ADP released from red cells can mediate SPA in shaken or stirred blood [13,14], if no precautions are taken [15]. Our method of SPA measurement described in the present study avoids these possible artefacts, and by using MEA instead of SPC (which requires fixation of blood samples at given time points), SPA can be continuously monitored [12]. 

Our results indicate that high SPA in blood as observed in a small percentage of human volunteers is due to platelet FcγRIIA stimulation by IgG immune complexes, as documented by the inhibition with antibodies against FcγRIIA and Fc of IgG, respectively. This might be explained by (a) a high reactivity of the platelet FcγRIIA to trace amounts of IgG immune complexes or (b) increased levels of circulating IgG immune complexes. 

Supporting the first possibility, platelet FcγRIIA expression was found to be increased in patients with cardiovascular diseases (acute myocardial infarction, unstable angina, or ischemic stroke) and interestingly also in healthy patients with two or more atherosclerosis risk factors [32]. Recently, expression of platelet FcγRIIA was proposed to be included in clinical risk scores to refine the assessment of cardiovascular risk after myocardial infarction [33]. It would be interesting to evaluate SPA according to the protocol described in our study in patients with cardiovascular diseases and evaluate its relation to platelet FcγRIIA expression. Furthermore, our data show a link of SPA and platelet aggregation induced by low-degree GPVI activation. Platelet reactivity to GPVI is enhanced in patients with diabetes and obesity [34,35], and platelet GPVI surface expression was significantly increased in patients with acute coronary syndrome, TIA, and stroke [36,37]. Our findings that Btk inhibitors abrogate SPA suggest that they might thereby reduce also enhanced platelet GPVI sensitivity to GPVI. Such a beneficial action might add to the suppression of atherosclerotic plaque-induced platelet GPVI activation by low concentrations of Btk inhibitors in vitro and ex vivo [18,19]. 

Our findings could also be related to the sticky platelet syndrome, a thrombophilic qualitative platelet disorder characterized by increased in vitro platelet adhesion and aggregation in response to very low concentrations of the weak platelet agonist ADP and/or epinephrine, and increased risk of arterial ischemic events [38]. This possibility deserves further investigation. However, our data did not show a link between SPA and platelet aggregation induced by another weak platelet agonist, CXCL12. 

A high platelet FcγRIIA reactivity might be also explained by a genetic polymorphism of FcγRIIA. Platelets from donors with the homozygous Arg-131-genotype are more sensitive to activating immune complexes [39]. In systemic lupus erythematodes (SLE), the FcγRIIA-Arg-131 genotype is overrepresented, associates with subclinical atherosclerosis [40], and platelets of homozygous Arg-131 SLE patients are more sensitive to stimulation as compared to SLE patients with the His/His-131 genotype [41]. Ligation of platelet FcγRIIA by immune complexes sensitizes platelets to activation by thrombin, which might explain vascular complications associated with SLE [42]. It would be of interest to study SPA in patients with SLE and its relationship to FcγRIIA genotype and vascular complications. Moreover, several Btk-inhibitors (fenebrutinib, branebrutinib) are being studied in clinical trials of patients with SLE. Inhibition of SPA by Btk inhibitors could be related to the clinical outcome. 

A further possibility underlying SPA may be the amplification of FcγRIIA-triggered aggregation by subthreshold concentrations of other platelet stimuli. These might particularly be stimuli activating the Gi-protein pathway in platelets [43] such as epinephrine [44] or CXCL12 [45] known to be present at low levels in blood. Additionally, the purinergic P2X1 receptor activated by ATP has been reported to amplify FcγRIIA-induced Ca^2+^ increases and functional responses of platelets [46]. Moreover, ATP as well as ADP might be released from red cells during the 10 min stirring period and could enhance SPA. However, our previous study showed barely any increase in ATP during 3 min of stirring using the described modified MEA protocol (see Figure 1D of [15]), and in the present study, we could not find significant inhibition of SPA by the PY12-receptor antagonist ticagrelor (data not shown). Furthermore, Btk inhibitors inhibited SPA but do not affect ADP-induced platelet aggregation measured by our modified MEA method [18]. Therefore, it is unlikely that ATP or ADP mediate SPA as measured in our study.

Our results show that ligation of FcγRIIA is required for high SPA. IgG-containing immune complexes must be involved, since increased SPA was inhibited by an anti-human Fc (IgG) antibody. The nature of the immune complexes can only be speculated about. Anti-PF4/polyanion IgG complexes were excluded in our study. They would have been a likely candidate, since in 5–7% of healthy blood donors anti-PF4/heparin IgG titers can be detected [28]. Rheumatoid factors (RF), which are autoantibodies of different Ig subclasses (IgM, IgG) against the Fc-portion of IgG and present in plasma from patients with rheumatoid arthritis and other autoimmune diseases, have been described in up to 5% of healthy 50-year-old persons [47]. However, evidence that RF/IgG complexes activate directly FcγRIIA receptors is lacking.

## 4. Materials and Methods

### 4.1. Materials 

The CD32 antibody IV.3 (monoclonal mouse IgG2b) was obtained from GeneTex (Irvine, CA, USA). The anti-CD32 antibody AT10 (monoclonal mouse IgG1) was from ThermoFisher Scientific (Waltham, MA, USA). Fenebrutinib (GDC-0853) was purchased from MedChem Express (Monmouth Junction, Middlesex, NJ, USA). Ibrutinib was from Selleckchem (Houston, TX, USA). Collagen was from Takeda (Linz, Austria). Dimethyl sulfoxide (DMSO) was from Sigma-Aldrich (Taufkirchen, Germany). Imbruvica capsules (140 mg) were from Pharmacyclics (Sunnyvale, CA, USA). The goat ImmunoPure^®^ anti-human Fc (IgG) antibody was from ThermoFisher Scientific (Rockford, IL, USA). PF4 was from ChromaTec (Greifswald, Germany). Heparin-Sodium was from Ratiopharm (Ulm, Germany). Abciximab (ReoPro^®^) was from Janssen Biologicals B.V. (Leiden, The Netherlands). 

### 4.2. Blood and Serum Collection

Venous blood was collected by cubital venipuncture from healthy donors who had not taken any antiplatelet drug for two weeks. A 21-gauge butterfly needle was used, and blood was drawn without venous stasis, into either 3 mL vacuum tubes from Dynabyte (Munich, Germany) containing hirudin (final hirudin concentration in blood: 200 U/mL) or S-monovette hirudin tubes from SARSTEDT (Nümbrecht, Germany) (final hirudin concentration in blood: 525 U/mL). The first 2 mL was discarded. The blood was kept at room temperature for 30 min, and measurements were performed subsequently within 2 h after venipuncture. 

S-monovette serum gel CAT/7.5 mL tubes (Nümbrecht, Germany) were used for serum preparation for the IgG-specific PF4/polyvinylsulfonate ELISA. After collection, the blood was kept undisturbed at room temperature for 30 min, followed by 1000× *g* centrifugation for 10 min at 4 °C. The aliquots of serum were stored at −20 °C before measurement.

### 4.3. Platelet Aggregation in Blood

Platelet aggregation measurements were performed by multiple electrode aggregometry (MEA) (Roche Diagnostics, Mannheim, Germany), which records the change of electrical impedance between two sensor electrode pairs caused by the aggregation of platelets, as described [12]. In brief, saline (300 µL) and aliquots (300 µL) of hirudin-anticoagulated blood were placed in aggregometer cuvettes (06675590, Roche, Mannheim, Germany). In order to avoid ADP leakage from red cells and subsequent ADP-mediated SPA, blood was first pre-warmed in the MEA cuvettes without stirring for 3 min, and then the stir bar was added and measurement was started [15]. Aggregation was recorded for 10 min. In some samples, fenebrutinib or DMSO (solvent control; 0.6 µL) was added and mixed well with a pipet, then covered and incubated for 15 min at 37 °C. Antibodies AT10 or IV.3 were added 3 min prior to the measurement. Platelet aggregation was recorded in arbitrary units (AU), and the area under the curve shows the cumulative aggregation (AU*min) from 0 to 10 min. The traces from a specific experiment whose values were closest to the mean were selected as representative traces.

### 4.4. Enzyme-Linked Immunosorbent Assay (ELISA) of Anti-PF4 IgG

The IgG-specific PF4/polyvinylsulfonate (PVS) ELISA (Immucor, Waukesha, USA) was performed to detect antibodies recognizing PF4/PVS in human serum, and the optical density (OD) values ≥ 0.400 are regarded as positive results per manufacturer’s instruction.

### 4.5. Statistics

The data are given as mean ± standard deviation (SD) of the indicated number of the experiments. The Shapiro–Wilk test was applied to assess the normal distribution of values. The Wilcoxon test was performed on the paired data and Mann–Whitney test on the unpaired data that did not pass the Shapiro–Wilk test. For normal distributed paired data, a paired *t*-test was performed. All correlations were calculated using the non-parametric Spearman test. 

## 5. Conclusions

In summary, we describe a novel protocol to measure SPA that uses stirred hirudin-anticoagulated blood and MEA. High SPA was detected in a small percentage of healthy donors. The underlying mechanism was dependent on platelet FcγRIIA stimulation by unknown IgG complexes and mediated by Btk activation. Future studies are warranted to investigate whether SPA as described in the present study might be a risk marker of atherothrombotic cardiovascular diseases and pathogenic factor accelerating SLE or other auto-immune diseases. 

## Data Availability

The raw data supporting the conclusions of this article will be made available by the authors, without undue reservation.
